# Radiofrequency ablation for autonomously functioning nodules as treatment for hyperthyroidism: subgroup analysis of toxic adenoma and multinodular goitre and predictors for treatment success

**DOI:** 10.1007/s00259-023-06319-9

**Published:** 2023-07-19

**Authors:** M. M. D. van der Meeren, F. B. M. Joosten, S. H. P. P. Roerink, L. N. Deden, W. J. G. Oyen

**Affiliations:** 1 Department of Radiology and Nuclear Medicine, Arnhem, The Netherlands; 2 Department of Internal Medicine, Arnhem, The Netherlands; 3 Department of Biomedical Sciences, and Humanitas Clinical and Research Center, Department of Nuclear Medicine, Humanitas University, Milan, Italy; 4 Department of Radiology and Nuclear Medicine, Nijmegen, The Netherlands

**Keywords:** Hyperthyroidism, Radiofrequency ablation, Thyroid, Thyroid nodule, Toxic adenoma, Ultrasound guided ablation

## Abstract

**Purpose:**

Treatment of hyperthyroidism caused by autonomously functioning thyroid nodules (AFTN) with ^131^I often leads to undesirable hypothyroidism. Radiofrequency ablation (RFA) has emerged as a promising alternative. This retrospective analysis aimed to examine the efficacy of, and postprocedural hypothyroidism after, RFA for AFTN.

**Methods:**

Patients with hyperthyroidism caused by AFTN and treated with RFA were included if follow-up of at least 1 year was available. Cure was defined as thyroid medication–free biochemical euthyroidism. To predict cure, patient and treatment factors were analysed. A distinction was made between solitary toxic adenoma (STA) and toxic multinodular goitre (TMG).

**Results:**

Forty-eight patients (36 STA, 12 TMG) were included. One year post-RFA cure rate was 72% in STA versus 25% in TMG (*p* = 0.004). One patient developed hypothyroidism. In 11 patients that remained hyperthyroid, a second RFA was successful in 83% of STA and 40% of TMG patients. At last available follow-up, this amounted to a total cure rate of 81% in STA and 33% in TMG (*p* = 0.002). In STA, cured patients had a higher baseline TSH and a lower FT3 than non-cured patients (*p* = 0.026 and 0.031). Cure was observed in 91% of patients when > 2.1 kJ/mL was delivered during RFA, compared to 44% when less energy was administered.

**Conclusion:**

The efficacy of RFA was nearly 3 times higher in STA patients compared to TMG. Severity of hyperthyroidism and kJ/mL delivered during RFA predicts cure. Direct comparison to the current standard of care is needed to implement RFA in treatment of hyperthyroidism caused by AFTN.

**Supplementary Information:**

The online version contains supplementary material available at 10.1007/s00259-023-06319-9.

## Introduction

Solitary toxic adenoma (STA) and toxic multinodular goitre (TMG) can cause hyperthyroidism through the presence of autonomously functioning thyroid nodules (AFTN). These AFTN produce thyroid hormone independent of regulation by thyroid-stimulating hormone (TSH). Excessive thyroid hormone production of AFTN induces TSH suppression, and may lead to overt hyperthyroidism over time. Hyperthyroidism caused by a STA accounts for approximately 5% of all causes of hyperthyroidism. TMG, harbouring multiple autonomous nodules, accounts for approximately 30% of hyperthyroid patients [1–3]. On thyroid scintigraphy, these autonomous nodules appear as regions with higher tracer uptake than surrounding thyroid tissue or so called “hot areas". In the case of concurrent hyperthyroidism, these nodules are commonly referred to as “toxic”.

Treatment of hyperthyroidism is indicated to reduce symptoms and risk of atrial fibrillation and osteoporosis in both overt and subclinical hyperthyroid patients [4–7]. At present, AFTNs are preferentially treated with radioactive iodine (^131^I) or alternatively with surgery. However, this treatment may lead to undesirable iatrogenic hypothyroidism. This leads to lifelong thyroid hormone substitution requiring regular laboratory monitoring. Hypothyroidism is associated with a lower quality of life, even when biochemical euthyroidism is achieved using thyroxine treatment [8, 9].

Although treatment with ^131^I is highly effective in curing the hyperthyroid status, the incidence of hypothyroidism is high. One year after ^131^I treatment, the incidence is 10–30% and increases to 30–60% 20 years post-treatment, probably because of prolonged radiation effects [6, 7, 10]. In order to avoid hypothyroidism, an alternative to ^131^I is warranted.

Radiofrequency ablation (RFA) is a minimal invasive, image guided procedure that may be an alternative treatment for AFTN. During RFA, an electrode conducts heat to thyroid nodules, resulting in cell death and nodular degeneration. This technique is already applied successfully to reduce mechanical complaints from nodular compression, since RFA decreases thyroid nodule volume by 77% 1 year post-intervention [11]. Recently, RFA has also been suggested as a treatment option for hyperthyroidism caused by AFTN. A systematic review and meta-analysis reported a success rate of 70% euthyroidism 1 year post-treatment without any cases of hypothyroidism [12]. The 2020 guidelines of the European Thyroid Association, recommend consideration of RFA for STA in young patients with small nodules [5]. However, the current level of evidence has not resulted in wide acceptance of RFA as a treatment option in these patients. Thus, there is a need to reproduce results in adequately designed and powered clinical trials, including extended follow-up periods. Predictive factors for success need to be further explored.

In addition, the relevance of differentiating between STA and TMG has not been unequivocally established. Therefore, our study aimed to evaluate long-term thyroid function and to assess patient and treatment-related factors influencing treatment success.

## Methods

### Patients

All patients treated with RFA at Rijnstate, Arnhem, The Netherlands, between July 2015 and December 2020 were included if they had hyperthyroidism caused by AFTN, and follow-up for at least 1 year was available.

AFTN was defined as hyperthyroidism caused by either STA or TMG. Patients with one single hyperactive nodule were categorized as a solitary toxic adenoma (STA) and patients with multiple hyperactive nodules as toxic multinodular goitre (TMG).

Preliminary results in a smaller group of the first 21 patients treated with RFA for AFTN were previously reported [13]. Here, we report a larger patient cohort with extended follow-up and subgroup analyses.

### Outcome definitions

The primary outcome measure was cure, defined as biochemical euthyroidism without using thyroid medication. Treatment failure was defined as either hypothyroidism or hyperthyroidism at any time point during follow-up at least 1 year after treatment. When a second intervention (e.g., ^131^I or another RFA) was chosen during the first year of follow-up because of persistent or progressive hyperthyroidism, it was also scored as treatment failure. Recurrent hyperthyroidism was defined as a period of euthyroidism after RFA, followed by hyperthyroidism.

### Laboratory testing, scintigraphy, and ultrasound

Hyperthyroidism was defined as a suppressed TSH level with elevated FT3 or FT4. Subclinical hyperthyroidism was defined as a suppressed TSH level with normal FT3 and FT4. Euthyroidism was defined as TSH within the normal range (0.3–5 mU/L) with FT3 and FT4 levels within the normal range (3.5–6.5 pmol/L and 12–23 pmol/L, respectively) without the use of any thyroid medication. Up until January 2019 the TSH assay by Roche was used (normal range 0.3–5 mU/L, analytical sensitivity 0.005 mU/L), thereafter the Atellica IM TSH3-UL ReadyPack by Siemens was used (normal range 0.3–5 mU/L, analytical sensitivity 0.008 mU/L). Hypothyroidism was defined as a TSH level > 5 mU/L, confirmed by repeated measurements at least 4 weeks apart without any thyroid medication. Graves’ disease was ruled out by a negative thyroid receptor antibody (TrAb) test. The diagnosis of AFTN was confirmed by combining thyroid scintigraphy and ultrasound. In most patients ^123^I scintigraphy was performed using the GE discovery 870 DR camera, 24 uptake scans were made after injecting 18.5 MBq of ^123^I intravenously. In a few patients, ^99m^Tc-pertechnetate was performed using 100 MBq of ^Tc99m^pertechnetaat and acquisition after 20 min. The presence of a hot nodule on scintigraphy had to correspond with a thyroid nodule visualised by ultrasound.

Nodule features were determined by ultrasound assessment according to the ACR-TIRADS [14], a well-established risk stratification system for thyroid nodules. In accordance to the ACR-TIRADS, nodules were scored on composition, echogenicity, shape, margin, and echogenic foci. The aim of using ACR-TIRADS was to describe nodules consistently but not to detect malignancy, since many toxic nodules would show characteristics of malignancy even though they are benign [15]. The volume of each nodule is calculated by *V* = *a* × *b* × *c* × 0.524 (where *V* is the volume in mL and *a*, *b*, and *c* the diameters of the ellipsoid. Nodules were classified as small (< 10 mL), medium (11–30 mL), and large (> 30 mL). Volume reduction of each nodule is calculated using the following equation: volume reduction (%) = ((baseline volume — follow-up volume)/baseline volume) × 100. The vascularity of each nodule was classified on pattern and intensity. The intensity was scored on a four-point scale: 0 (avascular), 1 (hypovascular), 2 (isovascular), and 3 (hypervascular). The pattern was also scored on a four-point scale: 0 (avascular), 1 (peripheral flow only), 2 (pre-dominantly peripheral flow), and 3 (pre-dominantly central flow).

### Radiofrequency ablation

Four radiologists with extensive experience with thyroid RFA performed all RFA sessions (FJ, PV, EB, and BT) [16]. All procedures were performed using a Viva RF generator (STARmed Seoul, South Korea) and an 18 gauge internally cooled electrode with a 7- to 10-mm active tip. The patient was placed in a supine position. For local anaesthesia, lidocaine 2% was injected subcutaneously and around the thyroid capsule. This also ensured hydrodissection, where fluid separates thyroid tissue from surrounding tissue to reduce complications caused by heat injury.

Following a previously described technique, the ultrasound-guided, trans-isthmic approach, and the moving shot technique were applied [17]. The target energy dose was > 2.109 kJ (0.5 kCal) per mL of nodal volume since this has been described as a threshold for optimal volume reduction [18]. The feeding vessel, as visualised by colour Doppler ultrasound, was not specifically treated. In TMG, the nodule was treated which was suspected to be most biochemically active, which would either be the largest nodule or the one with the most tracer uptake on scintigraphy.

### Data collection, analysis, and presentation

Data at baseline, 1 week, 3-, 6-, and 12 months post-RFA and all available subsequent follow-up data were retrospectively collected in Research Manager (cloud9 health Solutions, Deventer, the Netherlands). Analysis was performed using SPSS (25, version 4, IBM, Chicago, USA). The database was locked in February 2022.

Proportions are reported as numbers and percentages. Laboratory (TSH, FT4, FT3) and ultrasound parameters (nodal volumes and volume reduction) are presented as medians and ranges. We considered patient- and treatment-related factors as possible predictive factors for cure. To analyse this, we looked for differences in cure versus no cure 1 year post RFA for various factors. Patient factors were: age, gender, disease aetiology (STA versus TMG), baseline TSH/FT4/FT3, and nodule ultrasound features as described above. Treatment factors were RFA wattage, amount of energy delivered (per mL tissue), and reported completeness of ablation. We tested difference in proportions at different cutoff values of amount of energy delivered per mL tissue.

Differences in proportions were tested by the X-squared test. When counts were less than five, Fisher’s exact test was applied. To compare medians between groups, the Mann–Whitney *U* test or the Kruskal–Wallis test were used for two or multiple independent samples, respectively. All tests were two-sided, a *p* value below 0.05 was considered statistically significant.

## Results

Forty-nine patients were treated for AFTN with RFA. One patient was excluded from analysis because she refused further follow-up after reaching euthyroidism 7 months post-treatment. This resulted in 48 patients available for final analysis, 36 with a single hyperactive nodule (STA group), and 12 with toxic multinodular goitre (TMG group). Baseline characteristics are presented in Table 1. There were no significant differences between groups.

**Table 1:** Baseline characteristics

	STA group (*n* = 36)	TMG group (*n* = 12)	*p* value
Age (years)	55 (27–74)	57 (34–80)	0.694
Gender (female)	30 (83%)	11 (92%)	0.662
TSH (mU/L)	0.01 (< 0.005–0.290)	0.02 (< 0.005–0.450)	0.598
Overt vs. subclinical hyperthyroidism	17:19	5:7	0.738
Anti-thyroid drug use	1 (2.8%)	2 (16.7%)	0.085
Number of autonomous nodules treated	1	1 (1–3)	
Baseline volume (mL)	14.4 (1.3–59.4)	8.7 (1.0–26.7)	0.063
Vascular intensity			0.097
0 1 2 3	0 2 (5.9%) 7 (20.6%) 25 (73.5%)	1 (10%) 0 0 9 (90%)	
Vascular pattern			0.129
0 1 2 3	0 6 (17.6%) 11 (32.4%) 7 (50.0%)	1 (10%) 1 (10%) 1 (10%) 7 (70%)	

Results are presented as medians and ranges in brackets or number and percentage in brackets.

We administered a median energy of 2.5 kJ/mL (0.6 kCal/mL) in STA and 2.8 kJ/mL in TMG(0.6 kCal/mL) (range 0.29–7.1 kJ/mL). The median treatment time was 13 min (range 2–29 min). In 43 of 48 patients, the RFA procedure was complete as reported by the radiologist. Five RFA procedures were discontinued due to impaired visualisation of the nodule (*n* = 4) because of hematoma or localisation of the nodule and one because of intolerable pain. Two other minor bleedings occurred during RFA, which did not impair the procedure and required no further treatment. We did not observer any other complications.

An additional summary of data availability at all follow-up points is given in Online Resource 1.

Nodule volume reduction rate one year after RFA did not differ between groups and was 67% in STA and 74% in TMG, respectively.

### Treatment success for STA and TMG 1 year post-RFA

**Fig. 1:**
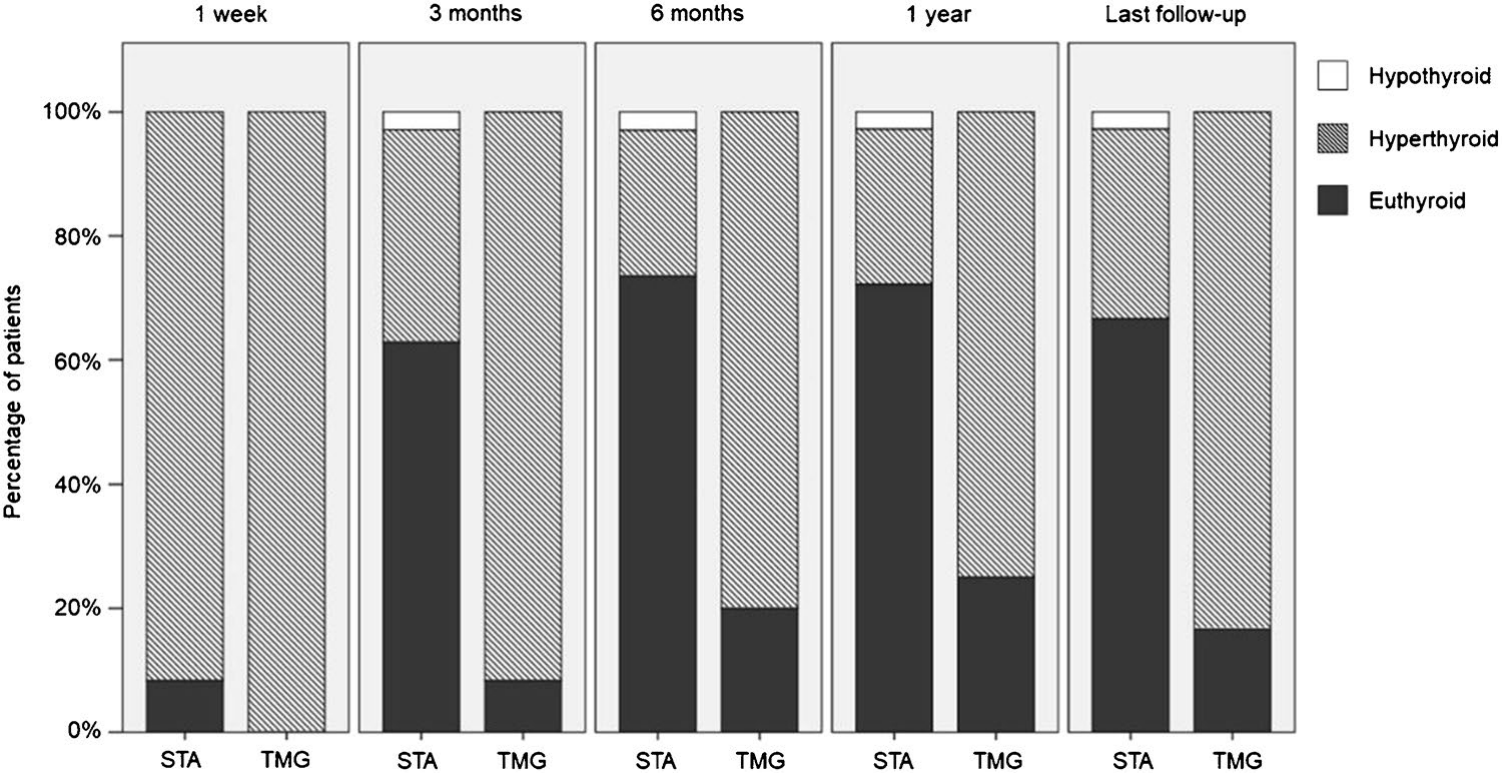
Stacked bar chart for thyroid function on different follow-up points after RFA for STA versus TMG

One year after the intervention, RFA was more successful in STA than TMG; 26 of 36 STA patients (72%) versus 3 of 12 TMG patients (25%) were cured of hyperthyroidism (*p* < 0.05). In AFTN overall, cure was achieved in 69% of patients. Figure 1 shows the number of patients and thyroid function status at each time point. The median time to cure was 3.1 months in STA and 6.5 months in TMG patients. In both groups, one patient had recurrent hyperthyroidism within the first year. One STA patient with a history of hemithyroidectomy developed hypothyroidism.

Of the three patients using ATD before RFA, none were treated successfully and required further treatment. However, ATD could be discontinued in one of these patients before retreatment.

### Follow-up after the first year

**Fig. 2:**
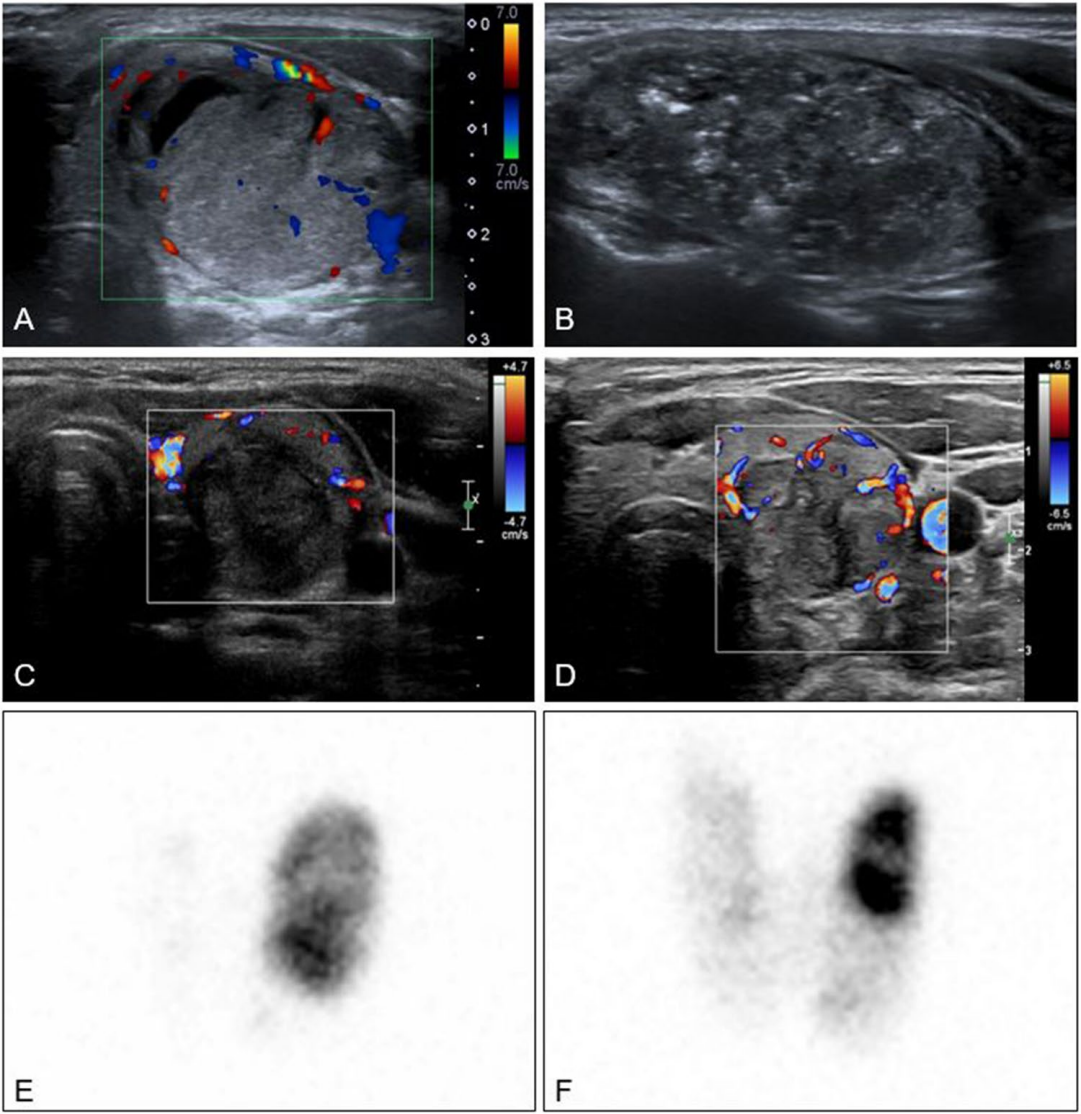
**A–D** Colour Doppler and grey scale ultrasound images of a patient successfully treated with RFA for STA. **A** Before RFA, showing a hypervascular nodule, volume 13.9 mL. **B** During RFA procedure with typical hyperechoic zones. **C** 4 months after RFA, volume 3.9 mL. The patient is biochemically euthyroid. No vascularity visible in treated zone but some vascularity in periphery of nodule. **D** 2 years after RFA treatment. Volume remains 4.0 mL and patient continues to be euthyroid. **2E-F** ^123^I scintigraphy image of thyroid. **E** Before RFA depicting a left sided hot nodule with near complete suppression of thyroid tissue. **F** 15 months after RFA. Reduction of volume and autonomy of left sided nodule and increased uptake of thyroid tissue

**Fig. 3:**
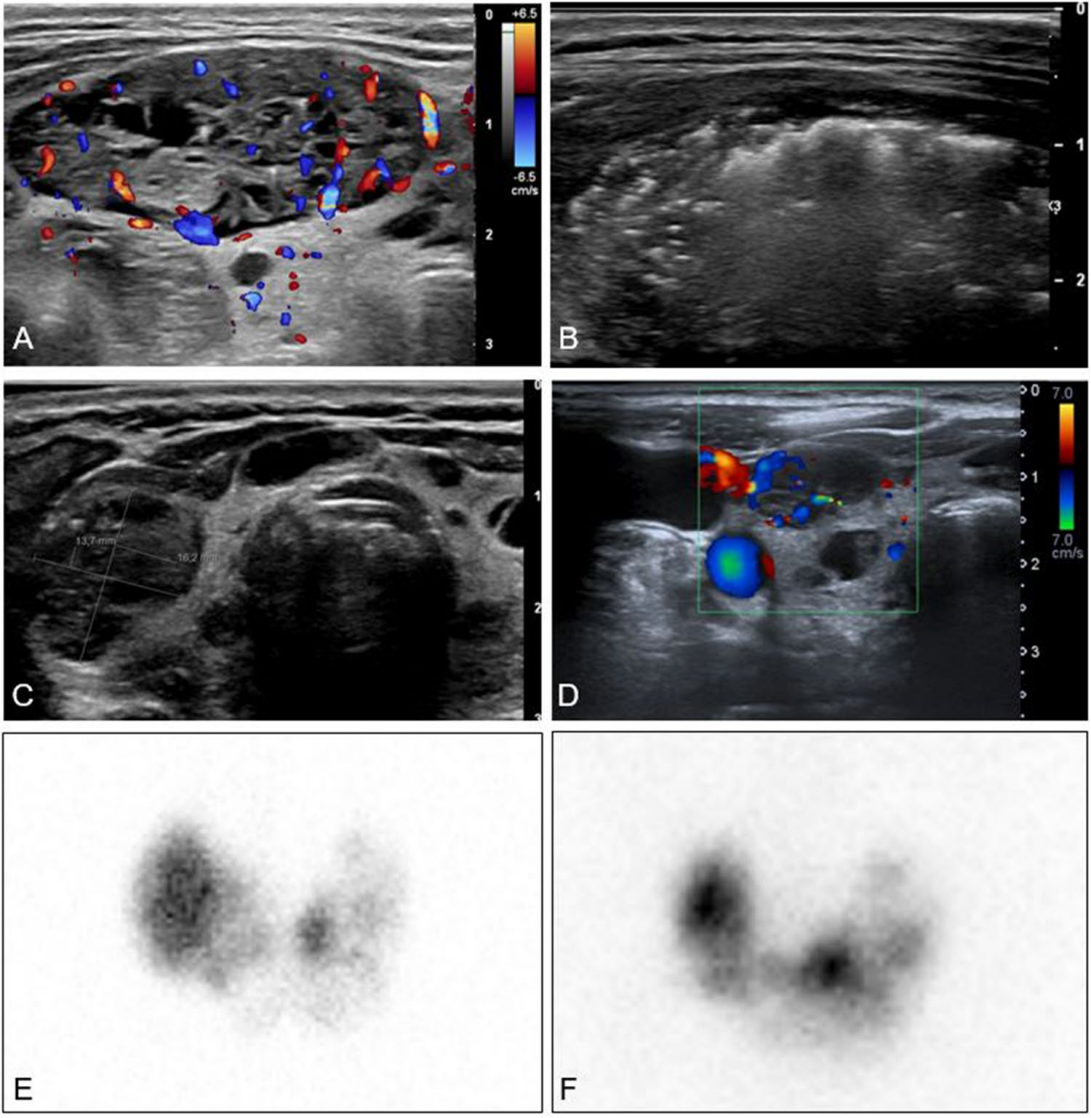
**A–D** Colour Doppler and grey scale ultrasound images of a patient successfully treated with RFA for TMG. **A** Before RFA, showing a hypervascular nodule, volume 8.9 mL. **B** During RFA procedure with typical hyperechoic zones. **C** 4 months after RFA, volume 2 mL. TSH has increased from below assay limit at baseline to 0.24 mU/L. **D** 1 year after RFA treatment, volume 1.1 mL. Reduction of vascularity of nodule but some vascularity in periphery of nodule. 2E-F Image of thyroid scintigraph using 123I as tracer. **E** Before RFA depicting inhomogeneous uptake with a right sided hot nodule. **F** 17 months after RFA. Reduction of volume and autonomy of right sided nodule and persistent look of inhomogeneous uptake

In the complete cohort, the median total follow-up period was 20 months (12–55 months). For 31 of 48 patients, intervention-free follow-up longer than 1 year was available. Two, three, and four years post-RFA, data on thyroid function and medication use were available for 21, 11, and 4 patients, respectively. One TMG (8.3%) and three (8.3%) STA patients developed late recurrent hyperthyroidism between 24 and 37 months post-RFA. This results in a cure rate after 1 RFA session of 24 patients (67%) in STA and 2 patients (17%) in TMG patients at the last available follow-up. Two case examples including ultrasound and scintigraphy of patients who were successfully treated are presented in Figs. 2 and 3.

### Predictive factors for success 1 year after 1 RFA session

To identify possible predictors for cure 1 year after RFA, we analysed patient and treatment-related factors. Since the TMG group was small, we only analysed these factors in STA patients.

Baseline TSH levels in cured patients (median 0.0195 mU/L, range: below lowest assay limit—0.29 mU/L) were significantly higher than in non-cured patients (median 0.009 mU/L, range: below lowest assay limit—0.014 mU/L *p* = 0.026,). Also, baseline fT3 levels were significantly lower in cured patients (median 6.2 pmol/L) than in non-cured patients (median 8.2 pmol/L, *p* = 0.031). No difference between cure and non-cure was found for age, gender, baseline fT4, baseline ultrasound features (nodal volume, the amount of cystic component in a nodule, vascularity, location of the nodule, and TIRADS) or VRR 1 year post RFA. Total energy delivered during RFA procedure also did not predict cure. However, in cured patients, a significantly higher energy delivery per mL of nodal volume was achieved as compared to energy delivery per mL in non-cured patients (median 2.7 kJ/mL (0.65 kCal/mL) vs. 1.6 kJ/mL (0.37 kCal/mL), respectively, *p* = 0.002). Cure was achieved in 20 of 22 patients (91%) where > 2.1 kJ/mL (0.50 kCal/mL) was delivered versus 5 of 10 patients (50%) where ≤ 2.1 kJ/mL (≤ 0.50 kCal/mL) was delivered (*p* = 0.02).

### Additional follow-up and re-treatment of failures

**Fig. 4:**
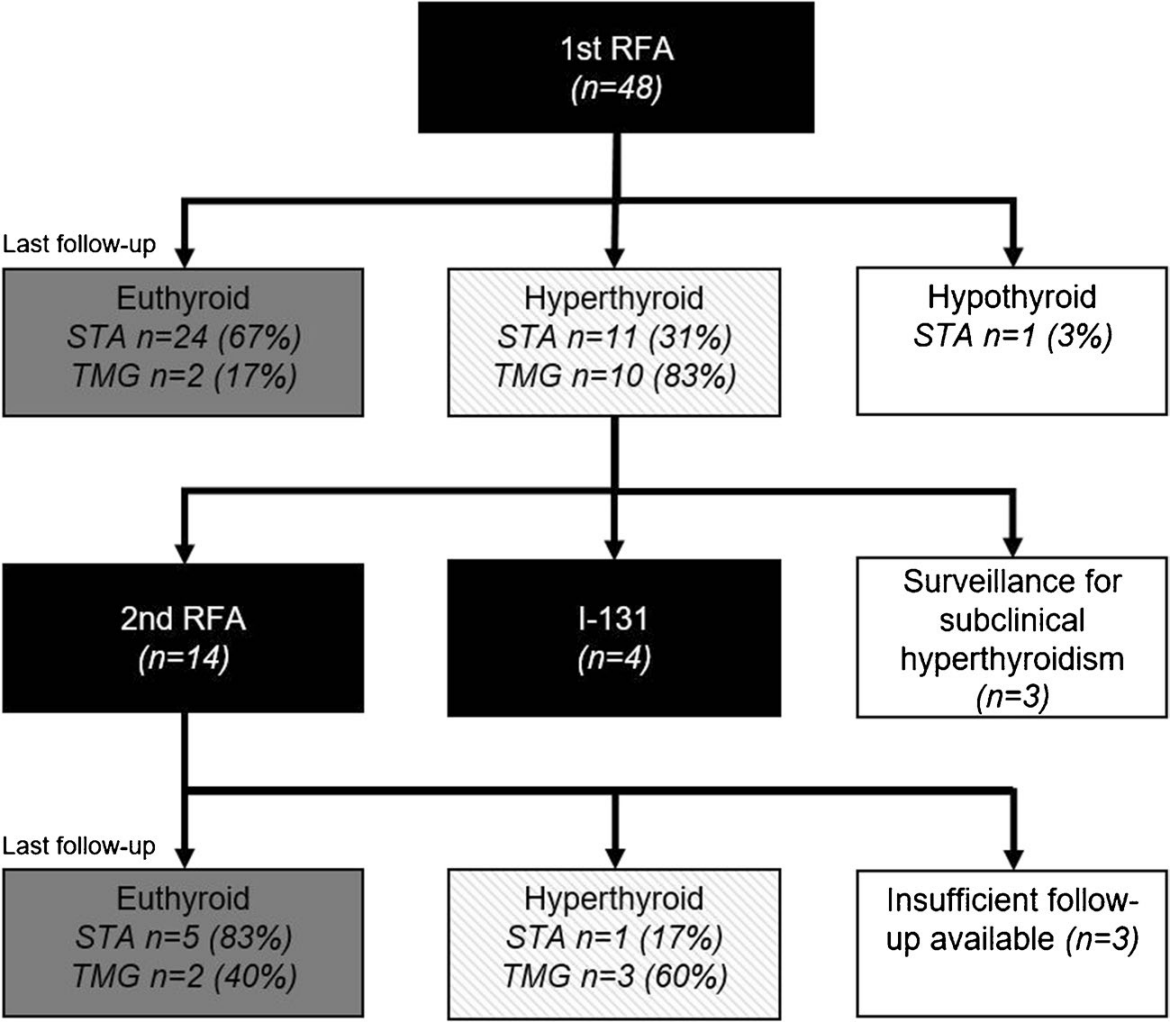
Flowchart showing follow-up and outcome after treatment for STA versus TMG

A flowchart showing the treatment course for each patient group is shown in Fig. 4. Of the 21 patients that remained hyperthyroid, three patients were on surveillance for subclinical hyperthyroidism, four patients underwent subsequent ^131^I treatment, of which one patient developed hypothyroidism, and 14 patients underwent a second RFA. Data of 3 of these 14 patients is incomplete because RFA had not yet taken place at time of data lock or, in one patient, a hurtle cell carcinoma was discovered in a new thyroid nodule for which a hemithyroidectomy was performed. For 11 of 14 patients that underwent re-RFA, follow-up of at least 1 year is complete. In these patients, a second RFA had a cure rate of 5 of 6 (83%) STA patients and 2 of 5 (40%) TMG patients at last available follow-up. This amounted to a total success rate of RFA after 1 or 2 sessions of 29 patients (81%) in STA and 4 patients (33%) in TMG at the last available follow-up. Overall, we achieved success in 34 of 48 (69%) of all patients with AFTN.

The STA patient who remained hyperthyroid received ^131^I and became euthyroid at final follow-up. Of the three TMG patients that remained hyperthyroid, two received ^131^I, and one of them became hypothyroid. The other received a third RFA but remained hyperthyroid and wishes no further treatment.

## Discussion

RFA appears a promising alternative to ^131^I as treatment of hyperthyroidism caused by AFTN [19]. However, experience is still limited, and little is known about predictors for treatment success and long-term effects. We retrospectively analysed a cohort of 48 patients who underwent RFA for hyperthyroidism caused by AFTN and analysed thyroid function, patient, and treatment factors that may influence treatment success over a follow-up period of 1 to 4 years.

This study confirms that RFA is feasible for treatment of hyperthyroidism in patients with STA. One year after RFA, 72% of patients became euthyroid, increasing to 81% at final available follow-up if a second RFA session is performed. The success rate was significantly lower in patients with TMG. In this group, 33% of patients were cured.

In this study, cure was achieved in 69% of all patients suffering from hyperthyroidism caused by AFTN after 1 or 2 RFA sessions. The current results are consistent with previous studies. A recent meta-analysis showed 69.9% TSH normalization after one or multiple RFA sessions without any occurrence of hypothyroidism [12]. We defined cure only as TSH normalization in the absence of thyroid medication, where some studies report patients as cured even when subclinically hypothyroid [20, 21].

In this meta-analysis, a higher amount of nodules than patients is reported, suggesting that both STA and TMG patients were analysed, but no distinction was made in the results [12]. This is the first study reporting results of RFA in TMG patients.

The difference in cure rate between STA and TMG might be explained by the difference in disease aetiology and the approach of RFA. In TMG, multiple autonomous nodules occur, but only one nodule at a time is treated with RFA. In this study, the difference in cure rate between STA and TMG could not be attributed to relevant differences in baseline or treatment factors. Since TMG is a much more frequent cause of hyperthyroidism, extending our knowledge of RFA in these patients is essential to determine whether there is an indication for RFA next to ^131^I in TMG.

To predict treatment success, we studied patient and treatment factors in STA patients. We observed that patients who were not cured after one RFA session had lower baseline TSH and higher fT3 values. Previous literature on this is limited. One study did not find a relationship between baseline TSH and cure using logistic regression [22]. In the previously mentioned meta-analysis, baseline TSH was not analysed as a subgroup for TSH normalization, but it was summarized in a table [12]. The studies reporting a higher proportion of TSH-normalisation reported a higher baseline TSH and vice versa [20, 21, 23–25]. This suggests that RFA might be less successful in more biochemically active AFTN. However, a more thorough and adequately powered clinical study is required to underpin the relevance of baseline TSH in predicting treatment success.

RFA is associated with mild to moderate pain and discomfort during the procedure. The reported complication rate is 2.2% [12]. We observed complications in 3 of 48 patients (6.3%), all related to minor bleeding during the procedural.

Large baseline nodule size has been proposed as a predictor for a less favourable result of RFA for both volume reduction and biochemical success [23], considering that a volume reduction of more than 80% is recommended [26]. In the current study, we could not demonstrate a relation between baseline nodal volume nor volume reduction rate and biochemical success 1 year after RFA. Earlier studies report conflicting results of baseline volume and treatment success. One study demonstrated a higher success rate 2 years after treatment in nodules smaller than 12 mL than in nodules larger than 12 mL (86 vs. 45%) [22]. In a larger cohort of 361 nodules a higher number of patients discontinued pharmacological therapy when nodules were < 10 mL at baseline and technical success was achieved as compared to patients whose nodules were larger than 10 mL and where technical success was not achieved [27]. However, another study in 30 RFA-treated AFTNs, did not observe a relation between baseline nodal volume and remission of hyperthyroidism [28]. An explanation for the variability of results may be another independent variable. In the current cohort, we observed a higher amount of energy delivered per mL tissue in patients who achieved biochemical success than those who remained hyperthyroid. However, many studies either do not report this parameter or only report the total amount of energy delivered, regardless of the nodule volume. In one prospective study, it was determined that when more than 2.7 kJ/mL (0.6 kcal/mL) during RFA is delivered, there is a 99% probability of achieving a volume reduction more than 50% [18]. In our cohort, we observed a significant difference in biochemical success when more than 2.1 kJ/mL (0.5 kcal/mL) was delivered. It is unclear if the difference between 2.1 and kJ/mL (or 0.5 and 0.6 kCal/mL) is clinically relevant. Given the inter- and intra-observer variability of measuring volume, a small difference in measured volume may lead to a large difference in calculated kJ/mL. We believe that aiming for at least 2.1 kJ/mL (or 0.5 kCal/mL) during RFA treatment is a reasonable strategy for increasing treatment success for both biochemical normalisation and volume reduction. To validate the relevance and applicability of this treatment parameter, future research should consistently report on energy delivered during RFA.

In addition to most studies, we reported follow-up beyond the first year after treatment [22, 29]. Extended follow-up was available in 31 of the 48 patients, with a median of 20 months (maximum 4.6 years). In addition, we observed four recurrences of hyperthyroidism. To our knowledge, this has not been reported before. However, as we do not have data at all timepoints for the complete cohort, this might be an underestimation of the total incidence of recurrent hyperthyroidism after RFA.

Currently, treatment with ^131^I remains the gold standard to treat hyperthyroidism caused by AFTNs. As hypothyroidism is a condition that requires lifelong treatment and follow-up, this should be taken into consideration when evaluating ^131^I treatment success.

One year after ^131^I treatment, the incidence of hypothyroidism is 10–30% which increases to 30–60% 20 years post-treatment [6, 7, 10]. One study retrospectively compared ^131^I to RFA in AFTN; they report cure in 18/25 patients (72%) after ^131^I and 20/22 patients (90.9%) 1 year after RFA. In the ^131^I group, 5 patients developed hypothyroidism and 2 patients developed subclinical hypothyroidism, compared to 2 patients with subclinical hypothyroidism in the RFA group [20]. In our study, hypothyroidism after RFA occurred in only one patient with a history of hemithyroidectomy. These findings support the hypothesis that RFA in AFTN may have a more favourable treatment profile compared to ^131^I, with a comparable rate of treatment success of hyperthyroidism with a lower incidence of iatrogenic hypothyroidism.

An alternative to treat hyperthyroidism caused by AFTN is surgery. In STA, this would lead to euthyroidism in most patients. However, surgery is not common practice in these patients due to higher complication risks and higher costs compared to ^131^I and RFA.

Limitations in our study are mainly related to its retrospective design with a limited number of patients. Follow-up data was not complete at all timepoints. Data on nodal vascularity, for example, was good at baseline (available for 44 patients) but scarce at follow-up (e.g., available for 20 of 48 patients at 1 year after treatment). Therefore, this dataset has limitations for identifying predictors that predict long-term success of RFA treatment.

A strength of the present study is the completeness of the pertinent data such as TSH, medication use, and nodal volume at baseline, which were available for all patients. TSH and medication use 1 year post-RFA were also available for all included patients. In addition, RFA parameters such as energy delivered were available for most patients.

Our data shows that RFA is a promising treatment option for patients with hyperthyroidism caused by AFTN, particularly STA. In TMG, treatment adequacy appears limited but number of treated patients is very low.

The risk of iatrogenic hypothyroidism after RFA appears low when compared to ^131^I treatment. The trade-off might be a lower cure rate after one RFA session, but after two RFA sessions, a similar result in curing the hyperthyroid state can be achieved. The costs of a RFA procedure are higher than ^131^I in an outpatient setting, due to the costs of RFA probes. However, to fully evaluate cost-effectiveness to healthcare of these two treatment options, the costs of inpatient ^131^I treatment and the costs of hormone substitution in case of iatrogenic hypothyroidism should be considered as well.

To implement RFA as treatment of AFTN-induced hyperthyroidism in clinical practice, data on long-term outcomes after RFA is needed since hyperthyroidism might reoccur after longer follow-up. To optimize the prediction of treatment success, the positive association between energy delivered per mL nodule should be confirmed. Also, a direct comparison of RFA to ^131^I treatment in a controlled setting with adequate follow-up and a formal cost-effectiveness analysis would provide essential additional insights.

### Supplementary Information

Below is the link to the electronic supplementary material.

Supplementary file1 (DOCX 15 KB)
