# Supplementary material for: Radiofrequency ablation for autonomously functioning nodules as treatment for hyperthyroidism: subgroup analysis of toxic adenoma and multinodular goitre and predictors for treatment success
Source: Eur J Nucl Med Mol Imaging. 2023 Jul 19;50(12):3675–83. doi: 10.1007/s00259-023-06319-9 (PMC10547644; doi:10.1007/s00259-023-06319-9)
Supplement: Supplementary file 1 — Supplementary file1 (DOCX 15 KB) [file 259_2023_6319_MOESM1_ESM.docx]

**Online Resource Table 1:
data-availability for 48 patients on different timepoints**

|  | Baseline | 1 week | 3 mnd | 6mnd | 1jr |
| --- | --- | --- | --- | --- | --- |
| TSH | 48 | 48 | 47 | 44 | 43^A^ |
| ft4 | 47 | 48 | 46 | 43 | 35 |
| ft3 | 45 | 42 | 41 | 33 | 31 |
| anti-TSH | 47 | x | x | x | x |
| Medication use | 48 | 48 | 48 | 48 | 47 |
| node volume | 48 | x | 43 | 42 | 44 |
| Vascularity | 44 | x | 24 | 22 | 20 |
| kCal delivered during RFA | 43 | x | x | x | x |

^A^ 4 missing values are due to re-treatment within the first year. 1 missing value is a patient who was euthyroid 6 months and 19 months after treatment
